# Supplementary material for: Are carbon emissions trading and green financial instruments synergistic? -Comprehensive quantitative research based on content analysis
Source: PLoS One. 2024 Mar 7;19(3):e0298601. doi: 10.1371/journal.pone.0298601 (PMC10919601; doi:10.1371/journal.pone.0298601)
Supplement: S1 Data — (ZIP) [file pone.0298601.s001.zip › Data/地级市Z_F工作报告.zip!/╣π╬≈/╖└│╟╕█╩╨/└┤╫╘╣1⁄2╓┌║┼í╛┬φ┐╦╩2╛▌═°í┐_2018.docx]

各位代表：

　　现在，我代表市人民政府向大会作政府工作报告，请予审议，并请市政协各位委员和列席的同志提出意见。

　　一、2017年工作回顾

　　过去一年，我们在自治区党委、政府和市委的坚强领导下，在市人大、市政协的监督支持下，紧紧依靠全市各族人民，贯彻新发展理念，坚持稳中求进总基调，以边海经济带建设为主线，着力推进六大经济业态发展，布局海湾新区建设，实施教育、卫生、脱贫三大攻坚战，城市知名度、影响力和综合竞争力显著增强，在广西区内保持总体增速领先、人均质量领先的发展势头，较好地完成市六届人大二次会议确定的目标任务。

　　主要工作和成效体现在以下几个方面：

　　（一）上级决策部署得到有效贯彻落实。认真贯彻党的十九大精神，坚持按习近平总书记对广西“五个扎实”的新要求指导各方面工作，防城港在国际通道、战略支点、重要门户建设中的地位日益上升。东兴试验区、跨境经济合作区建设加快推进，全国构建开放型经济新体制综合试点试验工作成效显著，一批改革创新经验在全国得到复制推广。延误近十年的防城港钢铁基地重新启动，生态铝工业基地顺利落户，临港产业在北部湾经济区中的地位日益凸显。精准脱贫攻坚取得新成果。构建大协同多锁链反走私防控体系的“防城港模式”向全国推广。自治区在我市召开“美丽广西?宜居乡村”服务惠民工作现场会。推行“1+4”绩效考评管理新机制，有力推动了自治区绩效考评指标的落地见效。

　　（二）经济发展稳中有进。坚持以发展经济为主线，积极落实稳增长的各项政策措施，多项经济指标排全区前列。全市GDP实现741.6亿元，增长6.7%；规上工业总产值1770亿元，增长20.1%，增速排全区前列，其中规上工业增加值362.3亿元，增长6.5%；固定资产投资672.8亿元，增长12.1%；财政收入74.5亿元；社会消费品零售总额124亿元，增长10.8%；外贸进出口总额113.6亿美元，增长29.4%，总额和增速保持排全区前列。城镇居民人均可支配收入32079元，增长7.8%；农村居民人均可支配收入13373元，增长10.4%。规上工业、固投、社会消费品零售总额、外贸进出口总额等权重指标均实现两位数以上增长，有力支撑了全市经济运行回升。

　　（三）重大项目建设实现新突破。组织实施重大项目攻坚行动，抓好项目保障，开展“招商季”活动，推动项目滚动发展。柳钢防城港钢铁基地正式开工，生态铝工业基地加快建设，作为“南向通道”重大交通设施项目的防东铁路和钦防高速公路改扩建工程开工，501号泊位建成验收，红沙核电二期、盛隆技改等一批项目稳步推进。布局建设海峡两岸产业合作区（防城港）产业园，整合组建防城港经开区，经开区规上工业总产值达到1273亿元，在北部湾经济区12个重点园区中排第1位，工业投资总量在全区113个园区中排第1位。防城港高新区获批自治区级高新区，建成我市首个自治区级院士工作站，签约入驻中检集团检验检测中心、中俄通用航空飞机总装厂等一批好项目，新增高新技术企业7家，每万人口发明专利拥有量增长率排全区第二。开工建设桂海冷链物流、川金诺化工等一批重大产业项目，枫叶粮油二期、澳加粮油膨化大豆粉等项目竣工投产。统筹推进“边海经济带”项目850个，完成投资472亿元。

　　（四）现代服务业升级发展。创新服务业供给方式，破解制约服务业发展瓶颈，加快建设防城港综合物流园、东兴国际综合性服务区等七个自治区级现代服务业集聚区，建成全国唯一联接东南亚国家的海陆冷链物流通道，铁路冷链物流成为全国发展示范标杆。成功入选国家首批农产品冷链流通标准化示范试点城市。东湾国家级示范物流园区及保税物流中心（B型）、集装箱办理站加快推进，粮食、煤炭等大宗货物“散改集”实现物流运输新模式。全市新增电商企业464家，实现销售额增长71.4%，东兴连续四年荣获“全国电商百佳县”，排名广西第一。房地产稳定健康发展，实现商品房销售面积239.6万平方米，增长9.2%。十万山森林生态游多点开花，白浪滩金滩滨海游日益火爆，跨境自驾游持续升温，东兴口岸通关人次达997万，“上山下海又出国”的旅游品牌更加响亮。第三产业增加值增长8.3%，超过GDP增速，三产占GDP比重提升0.5个百分点，产业结构不断优化。

　　（五）开放创新广度深度不断拓展。深入实施开发开放战略，主动融入“一带一路”和中新互联互通南向通道建设，开放型经济活力潜力加速释放。全国构建开放型经济新体制综合试点试验工作推进会在我市召开，防城港试点试验工作通过国家中期评估，“六大改革、六项探索”、34证合一“一照通”商事制度、“六个一”市场监管新模式、“企业登记全程电子化”等一批先行先试成果成为全国可复制推广的“防城港经验”。建立“四证两险一中心”跨境劳务管理模式，在广西率先开展了外国籍自然人经营户登记管理工作，东兴口岸获批扩大开放至北仑河二桥，为国际性常年开放公路客货运输口岸，峒中口岸（含里火通道）获批双边性常年开放公路客货运输口岸。实施加工贸易倍增计划，促进边境贸易转型升级。推进东兴试验区管理体制改革，加快东兴—芒街跨境经济合作区基础设施建设，北仑河二桥顺利建成，国门楼、标准厂房、验货场建设取得新进展，中越东兴—芒街互市便民临时浮桥建成开通。

　　（六）海湾城市特色更加彰显。实施“强中心”战略，高起点高标准推进海湾新区建设，投资150亿元新建100个海湾新区基础设施项目。有效处理一批道路建设历史遗留问题，江山半岛环岛东路、防城至江山一级路、文昌大道竣工通车，建成东湾大道、西湾大道和全海景马拉松赛道，海湾新区“五横五纵”主干路网正式打通。40公里的海湾绿道、西岸公园、城市沙滩等环西湾景观带项目建成使用，园博园、伏波大道开工建设，文昌大桥等项目前期工作基本完成。获批2017年度电信普遍服务试点城市，重点公共区域免费WiFi接入服务实现全覆盖。倡导绿色出行，成为广西沿海首个引进共享单车的城市。“七城同创”扎实推进，“城在海中，海在城中”的全生态海湾城市芳容初显。

　　（七）三农发展提质增效。深入推进农业供给侧改革，三农工作不断取得新成效。培育引进一批新型农业经营主体，创建各级农业示范区31个，东兴京岛海洋渔业获评自治区四星级示范区，东兴鑫宇金花茶产业和防城火龙果基地获评自治区三星级示范区。“十万山牛大力”获批国家地理标志产品，新认证3个富硒农产品，新增7个农产品“广西名牌”。创建8个宜居乡村示范村、4个助农增收生态养殖示范场、20个休闲农业点，发展农旅融合示范点80家。发展农业产业化经营组织417个。红树林海洋渔业核心示范区渔光互补项目成为“接二连三”发展新亮点。白龙珍珠湾海域海洋牧场一期项目建成。创建畜禽养殖标准化示范场国家级2家、自治区级2家。土地确权基本完成。宜居乡村专项活动深入开展，创新实施服务惠民“1+6+N”模式，推动农村基本公共服务全覆盖，农民群众办事不出村，有序推进改厨改厕等基础便民工作，规范农村垃圾收运处理，打造出一批有特色有亮点的乡村建设示范点。

　　（八）社会民生持续改善。始终把保障和改善民生作为政府一切工作的出发点和落脚点，财政民生支出占比达78.5%，投入96亿元实施一批为民办实事工程。扎实推进脱贫攻坚，贫困县“九有一低于”指标、15个预脱贫村“十一有一低于”指标均基本完成，全市2696户11310人脱贫，19个贫困村及上思县脱贫摘帽，2016、2017年易地扶贫搬迁项目综合进度均名列全区前茅。大力发展教育事业，北部湾高中、市理工职业学校一期投入使用，海湾新区10所新建学校加紧推进。推进义务教育均衡发展，港口区通过国家评估验收，东兴市、上思县通过自治区评估验收。深化医药卫生体制改革，落实药品零差率销售、医疗服务价格调整和医保支付政策改革工作。推进“区域医联体”及“县乡一体化”建设，建立医联体4个、专科联盟50个。市妇幼保健院迁建主体工程加快建设。推行生育全程医疗保健服务。提高低保保障标准，城市低保每人每月增加30元、农村低保每人每年增加250元。完成全民参保登记计划全国试点城市工作，社会保险覆盖面居全区前列。完成棚户区改造房2114套，危房改造竣工2143户。深入推进平安防城港建设，群众安全感满意度93.3%，提升幅度排全区第一。在全区率先基本实现气象现代化，基层防灾减灾救灾应急能力持续增强。安全生产形势稳定向好，食品药品专项整治成效明显。东兴“边境之窗建设工程”成为全国六大试点之一，国防动员、人民防空和后备力量建设、双拥共建等工作取得新成效。

　　（九）生态文明建设扎实有效。加强生态治理和保护，努力营造山清水秀自然生态，建立生态经济重大项目库，加快建设生态经济重点工程。全面落实“水十条”“大气十条”“土十条”，落实“河长制”，全市共设河长1440名。加快“蓝色海湾”整治，强化近岸海域、港口粉尘、建筑工地、河流湖库综合治理，加强北仑河口等重点区域的生态保护，红树林及沿海生态环境进一步修复，中央环保督察反馈问题整改得到较好落实。加强黑臭水体整治，完成生活污水直排口整治53个，10个乡镇污水处理厂基本建成。完成农村垃圾治理两年攻坚任务。全市集中式饮用水源地水质达标率100%，环境空气优良天数比例为92.9%，排全区第一。深入开展美丽防城港乡村建设和宜居城市建设活动，城乡人居环境显著改善。

　　（十）文化建设成效显著。加强文化体育建设，国家公共文化服务体系示范区创建通过国家中期评估。首届中国—东盟防城港马拉松激情开跑，来自15个国家和地区1万多名参赛选手同场竞技，中央电视台全程现场直播，赛事开创国内“五个第一”，荣获“最美赛道特色赛事”“铜牌赛事”两项国家级奖项。侨商广西行走进防城港，吸引了世界30多个国家和地区一大批有影响力的侨商侨领参加活动。举办海上丝绸之路防城港海洋渔业论坛、第二届中国—东盟商会领袖高峰论坛防城港分论坛暨物流业合作发展主题论坛、世界钦廉灵防同乡恳亲大会。市博物馆顺利开馆。广泛宣传黄永腾、黄炳坤等一批时代楷模。“欢乐西湾”“明江之夜”“边城欢歌”等系列群众广场文艺活动持续开展，采茶戏等地方戏曲得到发展传承。中越足球赛、国际龙舟赛、国际海钓邀请赛、“三月三”嘉年华、京族哈节、金花茶节、中越界河对歌等系列文旅节庆活动成为城市名片，城市的知名度美誉度进一步提升。

　　此外，外事侨务、海关、检验检疫、信访、人防、消防、档案保密、统计、质监、供销、粮食、通信、海事、金融、保险、地震、水库移民、农机、宗教、社会科学、决策咨询、地方志、工会、共青团、妇女儿童、老龄、残疾人、红十字、机关事务等工作都取得新进展。全市边疆稳定，民族团结，社会和谐，人民安居乐业！

　　一年来，我们深入推进“两学一做”学习教育常态化制度化，全面学习贯彻党的十九大精神，增强“四个意识”，不断加强政府自身建设，全面落实中央“八项规定”精神和国务院“约法三章”，不断转变工作作风。推进关键性改革，“三去一降一补”取得实质性进展，抓好大部制改革和执法体制改革，设立口岸管理委员会，完成公安系统大部制改革。开展相对集中行政许可权改革试点，组建市行政审批局，强化项目联合审批，完善政府部门清单制度管理。加快国有林场、集体林权、农村金融、供销社等改革。整合乡镇“七站八所”政务服务资源，实现政务服务便捷化。加强行政监察和审计监督力度，严肃查处各类违纪违法案件。加强法治政府建设，完善政府法律顾问制度，自觉接受监督，人大代表建议和政协委员提案办复率100%。推动管理创新，建立市、县、乡三级联网政府服务和政府信息公开统一平台，制定“学懂用”和“1+6”等各项制度，政府工作更加透明开放、扎实有效。

　　各位代表！过去一年，我们取得的成绩来之不易。这是自治区党委、政府和市委正确领导的结果，是市人大、市政协监督支持的结果，是全市人民团结奋斗的结果。在此，我代表市人民政府，向全市各族人民，向人大代表、政协委员，向我市驻军、武警官兵和中直、区直单位，向各民主党派、工商联、无党派人士、人民团体，向关心支持防城港发展建设的港澳台同胞、海外侨胞及海内外朋友们，表示崇高的敬意和衷心的感谢！

　　在肯定成绩的同时，我们也清醒看到发展中还存在着不少困难和问题，主要表现在：经济总量偏小，产业结构不优，新动能培育不足，质量效益不高；投入不够，稳增长基础不牢固，发展后劲不足；一些任务指标未能达到预期，港口优势未能充分发挥，财政收入质量还不够高；基础设施和公共服务供给不足，民生发展不平衡不充分的问题依然突出；政府工作效能有待提高，营商环境还需进一步改善等。我们要敢于正视问题，勇于担当，克难攻坚，百折不回，全力开创工作新局面。

　　二、2018年主要工作建议

　　2018年是贯彻党的十九大精神的开局之年，是我国改革开放40周年，是决胜全面建成小康社会、实施“十三五”规划承上启下的关键一年，是自治区成立60周年，也是我市建市25周年、建港50周年，做好今年工作意义重大。我们要全面贯彻党的十九大精神，以习近平新时代中国特色社会主义思想为指导，落实自治区党委、政府和市委的决策部署，坚持稳中求进工作总基调，坚持新发展理念，紧扣社会主要矛盾变化，按照高质量发展的要求，统筹推进“五位一体”总体布局和协调推进“四个全面”战略布局，坚持以供给侧结构性改革为主线，以“产业提升年”“项目提速年”“城乡提质年”为载体，以实施“三年行动计划”为抓手，大力推进改革开放，推动质量变革、效率变革、动力变革，加快构建现代化边海经济体系。加强和改善民生，促进经济社会持续健康发展，为着力营造“三大生态”、率先实现“两个建成”、奋力谱写新时代防城港发展新篇章奠定坚实基础。

　　今年经济社会发展主要预期目标建议是：地区生产总值增长保持在7.8—8.3%区间；财政收入增长6%，规模以上工业增加值增长8.5—9%区间，固定资产投资增长11%，招商引资到位资金增长11%，社会消费品零售总额增长10%，外贸进出口额增长12%，港口货物吞吐量保持亿吨水平，城镇居民人均可支配收入增长8%，农村居民人均可支配收入增长10%，居民消费价格指数涨幅控制在3%左右，城镇登记失业率控制在4%以内，能源消费总量和强度“双控”及温室气体排放、主要污染物排放量均控制在自治区要求范围内。

　　做好今年工作，一方面，我们要把工作目标任务放在自身需要与可能上来争取，保持较快较好的发展速度。力争实现速度与质量并进、人均与总量同步、总量与结构平衡、快发展与打基础兼顾、强优势与补短板并行，确保完成和超额完成预期目标任务。另一方面，我们要按照适应和引领新常态的要求，多做打基础利长远的工作，为今后更大发展奠定坚实基础。要围绕三年实现GDP超千亿，财政超百亿的“千百目标”和我市新时代“三步走”目标任务，对照“两个率先”指标体系，在不平衡不充分上补短板强弱项，在实体经济、乡村振兴、项目投资、城市建设、南向通道、现代服务业、教育提升、卫生健康等方面实现新突破。

　　做好今年工作，我们还要在“高、稳、进、新、活”五个关键方面聚焦发力。“高”即坚持高质量发展这个根本要求，大力推动质量变革、效率变革、动力变革，强化财政收入、居民收入、技改投资、高技术产业、研发投入、生态环境等指标。“稳”即坚持把稳增长摆在首位，重点是稳投资、稳工业、稳进出口、稳农业、稳消费、稳财政。“进”即坚持加快发展，形成新的增长点、增长极，在工业经济上求进，上新项目大项目；在城市建设和交通、港航等基础设施建设上求进，有更大的投入；在对外开放、乡村振兴、服务业、社会民生上求进，有更大的作为。“新”即坚持培育壮大新动能，从经开区传统优势产业转型升级中嫁接新动能，形成新的产品产值；从高新区新兴产业中培育新动能，形成新技术新业态；从乡村振兴中发现新动能，促进一二三产业融合发展，拓宽农民增收渠道；从完善港航投资营运体制和参与南向通道建设中壮大新动能，拓展新空间，搭建新平台。“活”即坚持盘活资源要素，优化营商环境，按照使市场起决定性作用、让政府更好发挥作用的要求，盘活土地资源，增加资金供给，缓解用工难题，深化国企改革，激活民间投资，推动放管服改革，营造良好环境，促进各种资源要素优化配置，释放经济活力。

　　各位代表！新时代要有新气象，更要有新作为。我们要坚持目标导向，强化“对标意识”，突出重点，统筹兼顾，着力做好以下工作：

　　（一）突出培育新动能，发展壮大实体经济

　　以实施“产业提升年”为抓手，把工业增量提质升级作为突破口，以高质量发展提升产业质量水平，把发展实体经济作为着力点，推动产业迈向中高端，做大做强实体经济，开启建设现代化边海经济体系新征程。

　　实施“实体经济倍增升级三年行动计划”。按照一年打基础、两年有突破、三年上台阶的要求，对工业发展目标、年度指标、重点产业、政策支持、要素保障、工作机制等进行科学安排。重点规划建设各类园区，加快重大产业项目建设。对接自治区沿海冶金工业“一核三带九基地”的布局，做大做强钢铜镍等支柱产业，推动冶金、糖、铝、粮油等产业二次创业向纵深发展。年内完成规上工业产值1965亿元，力争突破2000亿元。到2020年，工业总产值力争突破3000亿元，工业增加值突破600亿元。再经过几年努力，经开区产值达3000亿元，高新区产值达1000亿元。

　　开工建设大项目新项目。加强产业链招商，推动钢铁、铝铜、化工等产业项目落地，推动河北敬业570万吨钢铁、河北胜宝500万吨钢铁、防城港储能新材料产业园、金川镍钴新材料一期、中核恒通高端装备制造、卡威新型专用特种汽车、川金诺湿法磷酸、高新区云朗科技园二期、华银医学检验所、昭衍新药研发平台、敬业制药、晟宇通自保温砖、钢研锌业功能材料、中俄通用飞机制造厂一期、中节能光伏农业发电等29个项目开工建设。

　　抓好在建项目竣工投产。坚持优势优先重点突破，确保在建重点项目尽快竣工投产。加快推进柳钢环保减量搬迁、生态铝基地、盛隆冶金产业升级技术改造、红沙核电二期、景昇隆合金新材料、蛇纹石综合利用、聚馨麦芽生产线、联友重汽组装加工、金滩管业二期、印建奇新型建筑材料等17个重大项目建设。着力推进中海油LNG、盛隆石墨烯基海洋工程防腐钢材、科元ABS装置、渔光互补光伏发电、广西杭氧气体升级改造、中电一期节能减排升级改造、菌海铜渣镍渣综合利用、金川冶炼环保技术改造、壮传食品加工基地等30个项目竣工投产。推动中越北仑河二桥口岸综合服务区、高新区云朗科技园、沙潭江生态科技产业园研发中心、广西检验认证服务平台、中科院遥感技术平台投入运营。

　　推动重大工业项目前期工作，巩固提高现有产能。扎实推进钒钛新材料产业园、连通循环科技创新项目、华能东湾天然气分布式能源、川化硫酸钾及水溶肥、金龙达新材料船舶制造、国茗金花茶精深加工、正邦畜禽饲料、渔洲坪磷酸工业园搬迁等23个项目前期工作。随着宏观经济的企稳向好，工业产品价格回升，我市现有重点企业装置负荷率、生产开工率和盈利水平均有所提高，我们要应势而动，抓好关键性增长因素，推动盛隆、金川、核电、中电、大海粮油、惠禹饲料蛋白、华润水泥等一批现有企业满负荷生产，确保我市工业产值持续增长。做好柳钢冷轧、鼎业粮油、启鑫机械、凯玺有色等21家企业上规工作，形成新增产值100亿元。

　　打造重点产业园区。继续做大做强经开区、高新区，加快推进跨境经济合作区、江平工业园区、冲榄园区、冲仑产业园、上思工业集中区、台湾产业园区、生物医药产业园建设。对接自治区重点工业园区基础设施三年行动计划，完善园区各项配套设施，努力把经开区打造成为“中国制造2025”国家级示范区。高新区要全面实施深化改革加快发展三年行动方案，推进“八项行动”和“三项改革”，打造创新型特色园区。每个园区都要布局一批产业创新平台，开展政产学研合作，促进科技创新成果产业化。积极推动与甘肃、重庆、天津等省市合作建设飞地园区。

　　（二）突出发展现代服务业，培育拓展新业态新增长点

　　以打造服务业集聚区为抓手，补上服务业发展这块短板，提高服务业对经济发展贡献率。

　　加快发展现代物流业。推动开通防城港至济南、福州、郑州铁路冷链对流专列，开通天津、大连、营口等海产品冷藏箱海上运输航线，建设海公铁多式联运大通道。推进东兴进境水果指定口岸、LNG储运项目和口岸通关一体化综合服务中心建设，加快东盟农产品国际采购物流中心、桂海国际农产品冷链物流中心、大西南公路港、新港公铁海联运物流中心、东盟义乌小商品城建设。加快建设保税物流中心（B型）、中良仓储有限公司仓储物流等一批项目。推动化肥、硫磺、贵重金属、重晶石等大宗货物“散改集”。

　　加快发展旅游业。编制全域旅游规划，申报创建国家全域旅游示范区，推进跨境旅游合作区和边境旅游试验区建设，抓好江山半岛创5A景区和三块石海洋乐园、东兴国际旅游集散中心创4A级景区。加快三月三文化旅游度假区、南山长寿谷文化旅游度假区、东兴东盟民族文化村、广西职工劳模疗休养基地、中越边关牛大力乡村旅游区等一批项目建设。推动发展民宿、汽车旅游营地、海洋牧场海钓等旅游新业态。发挥沿海沿边沿山优势，大力发展乡村旅游，建设一批休闲农业与乡村旅游精品项目，打造滨海休闲、边关风情、十万山生态等乡村旅游带、精品旅游线路。

　　加快发展金融业。建设“政金企”对接信息平台，实现资金供需双方信息的充分共享，建立政银企联席会议制度，打造良好的政银关系、银企关系。围绕项目需求，综合运用贷款、基金、债券、融资租赁等金融手段做好实体经济融资服务。深化沿边金融综合改革示范区建设，拓展人民币/越南盾双币种现钞跨境双向调运业务。发挥好政府产业投资引导基金作用，放大资金杠杆效应。

　　加快传统服务业提档升级。深入实施第二轮“加工贸易倍增计划”，加快外贸转型升级。有序推进边民互市贸易升级发展，扶持边贸扶贫产业园等边境地区产业园区建设，重点抓好跨境经济合作区加工贸易，推动来料加工、装配业务和协作生产。发展市场采购贸易和跨境电商，引进阿里巴巴“一达通”等外贸综合平台。建设大型商业综合体和区域性消费中心，推动批发、零售、住宿、餐饮等传统商贸业集聚发展。做好服务业统计上规工作。

　　（三）突出实施乡村振兴战略，加快发展县域经济

　　以实施“乡村振兴三年行动计划”为抓手，坚持农业农村优先发展，推进农业供给侧结构性改革，突出绿色生态导向，发展特色产业，加强基础设施建设，增加公共服务有效供给，增强县域发展动力和活力。

　　实施“乡村振兴三年行动计划”。对接国家、自治区实施乡村振兴战略的统筹安排，加大资金投入，开展乡村产业发展、基础设施和公共服务设施能力提升专项行动，重点支持县域和乡村建设。实施一二三产融合、工业、旅游、健康、生态产业五大工程，大力发展特色产业。提升基础设施能力，实施交通、水利、城镇、能源、信息网络、园区六大工程，围绕镇镇通二级路和村屯通硬化路实施一批项目，推进“四好农村路”建设。提升公共服务设施能力，实施公共教育、卫生服务、文化体育、社会服务、就业服务五大工程，增加基层公共服务资源。力争到2020年县域地区生产总值达到1000亿元，4个县（市、区）财政收入都超过10亿元，港口区进入全区城市主城区前列，东兴市进入全区重点开发区前列，防城区进入全区重点开发区的上游水平行列，上思县进入全区重点生态功能区的上游水平行列。

　　夯实农业农村工作基础。落实农村土地“三权分置”制度，抓好农村集体产权制度改革，全面完成农村土地确权，推动土地流转，发展适度规模经营。抓好土地开垦和旱改水工作，落实耕地占补平衡。强化农村建房审批。抓好农村金融改革和政府平台建设，成立市、县级农业投资公司，设立农业投资发展基金，增加农业发展的资金支持。引进和培育农业龙头企业，增强龙头企业带动引领功能。加快农村道路、互联网、饮水供水设施、水利水电设施、高标准农田等基础设施建设，完善村庄生活垃圾收运处理系统，改善农村生产生活条件。

　　以推动现代农业示范区增点扩面提质升级为重点，发展县域特色产业。要建成自治区级示范区4个、县级9个、乡级32个，争取创建1—2个国家级现代农业产业园。全市完成农林牧渔业总产值163亿元，增长4.5%；农民人均可支配收入14710元，农业固定资产投资完成150亿元，农业招商引资突破200亿元，休闲农业总产值达5亿元。建成2.5万亩“双高”糖料蔗基地，特色水果总产量达到7万吨，蔬菜稳定在30万亩左右，茶叶产值2亿元以上，推动扩大茶叶种植面积，金花茶、白石牙茶等品牌影响力不断上升，培育一批国家地理标志农产品和区域品牌，富硒农产品面积达到1万亩以上，牛羊猪规模化、生态化养殖比重达70%以上。规划建设“边海?京韵”“边海?珍珠湾”等一批田园综合体，从白龙珍珠湾沿岸到东兴竹山古榕部落，整体规划建设一批村庄，提升一批乡村基础设施，美化亮化绿化一批村庄，建成一批一二三产融合发展示范点。

　　以发展农产品加工为重点，做大县域经济总量。以农产品加工园区为载体，推进特色农业发展，延伸农业产业链，加快补齐农产品加工短板。强化产地加工，推动农产品加工向工业园区集中。加快推进东兴跨境水产品加工区、防城边贸商品加工区、上思农产品加工区、冲榄工业园水产品加工区、企沙中心渔港水产品加工区建设，打造面向东盟的农产品加工基地。每个县（市、区）都要有园区的支柱产业来支撑，重点打造1—2个优势特色产业，提高工业经济对GDP的贡献率。

　　以县域城镇化为重点，集聚发展要素和人气。县域城镇化是新型城镇化的重点，是推动城乡一体化发展的关键。要加强城镇土地储备，为加快县域城镇化建设提供有力的用地保障，规范土地一级开发运作，依法营运公共资源。创新县域融资产品，支持县（市、区）有条件的企业开展资产证券化、发行企业债券等融资，鼓励设立小城镇发展、产业投资等基金。规范整合县级政府投融资公司，开展县域基础设施PPP投融资模式，推进交通、电商物流、小街小巷改造、城镇配套工程等一批城镇项目建设。加大城乡统筹力度，推动农业转移人口市民化，为进城农民安居乐业做好保障工作。

　　发展现代海洋渔业。发展生态渔业和设施渔业，加快养殖池塘标准化建设和近海网箱设施改造，全年养殖池塘标准化改造面积5万亩以上，深海抗风浪网箱养殖发展到120万立方米，陆基工厂化养殖发展到10万平方米。规划建设企沙中心渔港经济区，启动东兴天鹅湾渔港经济区、防城区双墩渔港经济区项目建设。继续抓好白龙珍珠湾海域国家级海洋牧场示范区建设。

　　持续开展美丽防城港乡村建设。完成宜居乡村建设，启动幸福乡村建设。开展农村人居环境整治三年行动，抓好农村危房改造，全面推进农村“厕所革命”，抓好改厨、改畜圈等工作，实现农村无害化卫生厕所、清洁厨房普及率达到自治区的要求。按照“产业兴旺、生态宜居、乡风文明、治理有效、生活富裕”的要求和全域防城港的理念，逐步探索开展村屯规划，抓好宜居乡村、幸福乡村示范点建设。

　　（四）突出加快新型城镇化建设，推动全域防城港发展

　　以“城乡提质年”为抓手，以新型城镇化建设为引擎，促进产城融合、城乡一体、全域发展，努力探索一条具有边山海特色的新型城镇化道路。

　　加快海湾新区建设。继续推进“中心区建设五年行动计划”，加快“堤路园”建设，确保到2019年上半年完成广西园林园艺博览会承办任务。开展防城江东岸开发前期工作，抓好文昌片区征迁安置，加快棚户区改造。建成江山大道西段、纬二线西段、北站南路西延长线等一批城市主干道，织密行政中心区、城南片区、高铁北站片区路网。加快打通一批断头路，逐年抓好路网主干线“白改黑”沥青化改造。坚持规划引领，供水、排污等基础设施建设先行，制订西湾西岸、防城江西岸的土规和城规，编制重点区域控制详规和城市设计专项规划，加快广投、名都、体育小镇建设步伐，完成长榄岛土地整理、海域清淤，同时开展招商和建设前期工作。抓好恒大悦珑湾、碧桂园滨海城等精品楼盘开发建设，推动房地产转型升级健康发展。

　　拓展新型城镇化新领域。按“产业向东，港产城融合；城建向西，点线面推进”的思路谋划新一轮城镇化。按“一带多组团、一组团一专业小镇、一片区一项目”的格局，编制江山半岛至东兴沿海沿江沿国门大道的新型城镇化总体规划，修订完善江平镇、江山镇控制性详细规划，打造珍珠湾区滨海特色城镇群。积极推动客天下?防城港东盟特色小镇、京族海洋特色小镇、金花茶特色小镇建设。按照“前岸中区后城”模式，促进东兴城区与跨境经济合作区互动融合发展。推动防城城区南联西移，有效疏解老城区的拥堵和低品位开发问题。推进上思县城建设提档升级。抓好企沙渔港经济区和经济发达镇管理体制改革。推动海湾新区与高新区、经开区连片融合发展，形成港产城互动发展新格局。

　　强化城市规划和市政管理。加快城市总体规划修编工作，优化城市发展空间布局。抓紧制订重点片区的控制性详规和城市设计，加强规划执行的刚性约束，提高城市品位。深入推进城市管理执法体制改革，理顺执法关系，改进执法方式，进一步明确城市管理的职责划分，加大市容环境综合整治力度，打造舒适优美的生活环境。加快智慧城市建设，优化交通、卫生、城管、园林、旅游等领域智慧应用系统。协调推进“七城同创”，争取年内国家园林城市、森林城市、卫生城市创建取得新突破。

　　（五）突出抓好项目建设，扩大有效投资

　　以推动交通、港航和城乡基础设施建设为抓手，以“项目提速年”为载体，加大投入，开展城乡基础设施建设大会战，增强发展后劲。

　　把项目工作作为扩大有效投资的切入点和基础平台。狠抓项目机制、储备、招商、落地四个环节，执行项目工作1+6制度，各县（市、区）、每个经济部门都要有储备项目库，强化项目精准招商，突出产业链招商、特色产业集群招商，解决好项目落地难落地慢落不了地的问题，打通项目落地“最后一公里”。实施“六个一百”项目攻坚，工业升级类、现代服务业类、城市建设类、社会民生类、交通港航类、乡村振兴类均要上100个项目，总投资3780亿，今年重大项目投资力争突破500亿。

　　抓好项目前期工作。把重大项目前期工作做深做细做实，提高项目成熟度和可批性。争取我市更多重大基础设施建设项目纳入国家和自治区项目计划盘子。今年要重点做好沙企一级路改扩建、东兴机场、东湾大桥、市体育场（馆）、高校园区、吴圩经上思至东兴高速公路、企沙南作业区、赤沙作业区等重大项目的前期工作。

　　加快重大项目建设。推进防东铁路、钦防高速扩建、企茅路、国门大道等一批交通基础项目建设，抓好储能材料、合金新材料、铝产业加工、新型专用特种汽车产业等产业项目建设。积极推动经开区、高新区、冲仑产业园等园区基础设施PPP项目建设。

　　做细做实城乡基础设施建设项目。聚焦我市城乡交通薄弱环节，实施县域路网攻坚工程。三年内计划建设总投资141亿元总里程1162公里的县域路网。年内开工建设4条县域对外动脉通道、5条县域重要交通节点互通循环公路、30条联接县域内部的县乡村道路，总投资72亿元。完善县域客运网络，发展县域特色物流。提升公共服务设施能力，年内投资13亿元，实施文化体育、社会服务等15个专项68个项目。争取国家、自治区兴边富民专项资金支持，组织开展边境地区0—20公里农村基础设施建设 。

　　（六）突出打好脱贫攻坚战，着力补齐民生短板

　　以继续推动脱贫、教育、卫生健康攻坚和为民办实事为抓手，坚持以人民为中心，在发展中补齐民生短板，更好满足人民美好生活的需要。

　　打赢脱贫攻坚战。整合扶贫力量，完善扶贫机制，强化脱贫监督问责，突出产业支撑、易地搬迁、贫困村集体经济发展、兜底保障、基础设施建设、干部帮扶等重点工作，围绕脱贫标准精准施策，着力提升教育、医疗、住房保障水平。向十万大山山区等深度贫困地区聚焦发力，把扶贫和扶志、扶智结合起来，激发贫困人口内生脱贫动力。年内完成脱贫人口5810人，16个贫困村脱贫摘帽。

　　下大力气补上教育事业短板。对接自治区教育提升三年行动计划和“十百千万”学校建设，落实好教育设施专项规划，办好学前教育、特殊教育，提高高中教育质量，新建改扩建幼儿园5所、义务教育学校10所，确保北部湾高中年内竣工。抓好义务教育均衡发展，争取防城区通过自治区评估验收，东兴市、上思县通过国家评估验收。加强教师队伍建设，引进一批名师名校长，培养一批学科带头人、中青年骨干教师。加快产学融合，发展综合职业技术教育，推动市理工职业学校与企业联合办学。高起点谋划发展特色高等院校，积极争取与国内外知名学府联合办高校，努力打好我市教育翻身仗。

　　下大力气夯实卫生健康事业发展基础。认真实施自治区基层医疗卫生机构能力建设行动计划，开工建设市第一人民医院迁建项目，加快一批乡镇、社区卫生服务基础设施建设。深化医药卫生体制改革，推进医联体建设发展。加快城市公立医院综合改革。预防控制重大疾病，深入实施防治艾滋病攻坚工程，抓好重大传染病和慢性病防治。做好自治区卫生城市复评审工作。落实全面两孩政策，打造生育全程基本医疗保健服务链条。推进医养结合，发展健康产业。

　　促进文化事业产业新发展。抓好创建国家公共文化服务体系示范区验收工作，推进市非遗馆、市美术馆、市体育中心等场馆建设。深入开展传统诗词、戏曲进校园、进社区、进农村活动。举办首届北部湾开海节，打造独具特色边海文化新品牌。办好中国—东盟国际马拉松、海上国际龙舟赛、中国—东盟国际海钓、帆船帆板赛、山地自行车赛“五大赛事”，提升京族哈节、金花茶节、“三月三”嘉年华等文旅节庆活动档次水平，不断擦亮我市文体品牌。落实自治区和市委部署，抓好自治区成立60周年大庆以及防城港建市25周年、建港50周年各项活动。

　　加强就业和社会保障。加快创业孵化基地、众创空间、农民工创业园建设，加强对灵活就业、新就业形态的支持。继续实施大学生就业促进和创业引领计划。做好征迁失海失地农民就业安置，开发乡村公益性岗位安排就业，支持企业在乡村创建扶贫车间、加工点，组织农村劳动力从事居家就业和灵活就业。推进全国社会保险标准化“先行城市”建设，拓展社保卡覆盖地域和应用范围。强化养老服务，做好孤儿救助，依法保障妇女、未成年人和残疾人的合法权益，筑牢社会救助体系。

　　创新社会综合治理。树立安全发展理念，落实安全生产责任制，坚决防范较大以上安全事故。做好自然灾害预警防治，提高防灾减灾救灾应急能力。加强寄递渠道安全管理。强化食品药品监管，力争获批自治区食品安全示范城市。依法严厉打击各类违法犯罪活动，做好禁毒与反走私、反恐工作，开展扫黑除恶专项斗争，推进信访工作规范化法制化，加快档案信息化建设，加强网络安全，提升群众安全感和满意度。发挥好工会、共青团、妇联等群团组织联系群众的桥梁纽带作用。加强国防动员和后备力量建设，强化边海防管控，做好双拥优抚安置工作。推进兴边富民行动，创建民族团结进步示范市，推进宗教信息化建设全国试点，建设幸福美丽和谐边疆。

　　抓好为民办实事。在统筹推进自治区人民政府为民办实事工程各项工作任务的同时，要认真落实本次人代会决议，倾心为民办好十件实事。我们要把老百姓的安危冷暖时刻放在心上，用感情倾听群众心声，了解群众疾苦，认真办好办实群众关心、百姓所需要的实事，让人民群众享有更多的获得感，生活一年更比一年好！

　　（七）突出建设南向海陆通道，构建开放型经济新体制

　　以实施“南向海陆通道三年行动计划”为抓手，深度融入国家“一带一路”建设，形成全方位多层次对外开放新格局，打造高层次开放型经济新体制城市。

　　实施“南向海陆通道建设三年行动计划”。主动参与广西加快推进中新互联互通南向通道建设工作，并结合我市实际制订三年行动计划。积极推进建设南向通道沿线铁路、公路、海港、口岸、物流园区等基础设施，重点建设东湾多式联运基地、港航服务中心，形成以海铁联运为主，以公铁联运、公海联运、跨境公路运输为辅的多式联运体系，努力将我市建设成为连接东盟国家重要的国际海陆贸易新通道。加快铁路集装箱办理站、综合交通枢纽等项目建设，力争开通防城港至香港班轮“天天班”。完成国际贸易“单一窗口”全覆盖，建成多式联运网络综合信息平台，压缩装卸货、提离货等环节时间。推动与越南等东盟国家“两国一检”试点。通过三年努力，防城港在南向通道中的影响更加凸显，独特的海陆通道新优势得到更充分发挥。

　　坚持以港兴市，建设向海通道。着眼于大港口建设，提升防城港集疏运能力。加快渔万港区511—512号泊位和513—516号泊位、榕木江西港点1—6号泊位等续建项目建设，力争开工建设东湾潭油航道、钢铁基地20万吨级3号码头。推进40万吨级码头及航道、潭油作业区5—7号泊位、云约江作业区17号泊位等重点港口项目前期工作，尽快启动赤沙作业区钢铁基地码头20万吨级1—2号泊位、一个10万吨级泊位和1.8公里岸线码头规划，具备条件的抓紧年内开工建设。利用国务院新近批复防城港口岸扩大开放的契机，加快完善相关基础设施和监管设施，加快解决十余年来困扰我市临港产业和口岸经济发展瓶颈问题。改革港口投融资模式，积极推进由政府主导向市场主导转变，开展港口投资和运营大招商，鼓励推动香港、天津和西南省市参与我市港口建设营运。积极开展从产业驱动物流发展到物流驱动产业发展模式的新探索，采取园区+产业+港航运输新模式，依照市场行为，鼓励有实力的多元主体开辟和营运新航线，加快发展向海经济。

　　坚持增点扩面，建设向边通道。规划建设东兴铁路口岸，抓好东兴口岸、峒中口岸基础设施建设。启动里火口岸升级改造，加快中越峒中—横模大桥的建设，开工建设G219防城峒中至东兴公路，推动中越里火—北峰生大桥建设。积极发展防城区边民互市贸易，形成口岸经济新的增长点。加快完成北仑河二桥口岸综合服务区一期配套工程、二期工程、口岸通关智能化工程及一批通关道路建设，尽早开通使用北仑河二桥。启用东兴—芒街北仑河临时浮桥，实现东兴边境贸易的提档升级，改变东兴“货少人多”的现状，促进人货两旺。要用足用活东兴试验区政策，拓展边民互市贸易转型升级、境外劳务合作等政策效应。利用国务院新近批复扩大开放东兴公路口岸、对外开放峒中（含里火通道）公路口岸的契机，探索开展国际性常年开放公路客货运输业务和双边性常年开放客货运输业务。加强中越口岸公路、铁路等互联互通的规划建设对接，推动中越跨境公路运输规模化运行，加密中越跨境公路货运直通车，规划建设跨境物流基地，培育多式联运龙头企业和企业联盟，大力发展口岸经济。

　　搭建开发开放平台载体。积极参与中国—东盟信息港建设，发展面向东盟的特色大数据产业，支持九次方大数据信息集团落户防城港，培养聚集一批开展数据分析、提供数据服务增值服务的企业。加快东兴试验区、跨境经济合作区、沿边金融改革试验区、边境经济合作区、跨境旅游合作区、海峡两岸产业合作区建设，深入推进开放型经济新体制试点建设，加快在边境口岸复制推广国检试验区创新措施，打造东兴—芒街商贸旅游博览会、中国—东盟海洋渔业论坛升级版。在交通、产业、政策等方面主动对接粤港澳大湾区和中新互联互通南向通道建设。

　　（八）突出加强环境保护，发展生态经济

　　以建设“美丽防城港”为抓手，大力发展生态经济，推进资源节约集约利用，打好污染防治攻坚战，加强生态环境保护建设，擦亮“山清水秀生态美”金字招牌。

　　加快发展生态经济。坚持把绿色发展作为调整经济结构、转变发展方式的重要途径，深入实施生态经济重点工程，在生态产业、生态基础设施、生态环境治理、生态城乡建设等领域推进一批项目建设。加快传统产业生态化改造，促进水产渔牧畜禽规模化养殖场生态化建设。强健生态经济之体，推进节能环保产业园和生态循环养殖产业园建设，支持经开区、高新区等新建生态产业园区提档升级。

　　抓好环境保护治理。全面落实中央环保督察反馈问题整改，深入实施大气、水、土壤污染防治行动计划，继续推进防城江、明江、北仑河等流域治理。抓好国家海洋督查问题整改，加强海洋环境保护，抓好近岸海域综合治理，落实围填海规划与管控措施。加强危险废物监管和安全处置，严厉查处倒卖、非法加工利用进口固体废物等违法行为，禁止洋垃圾、危险废物进入我市。完成第二次污染源普查和第三次土地调查。加强天然林、生态公益林管护，调整水源地、景区周边的森林树种结构。全面推行河长制，探索实行湾长制，确保“水清岸绿河畅景美”。实施自然资源资产离任审计制度，坚决守住“生态红线”。打好生态环境保卫战，抓好农业面源污染整治和城市黑臭水体治理，推进海域海湾生态修复工程，加强港口码头和工业粉尘治理，保持空气环境质量排全区前列，坚决守护“防城港蓝”！

　　（九）突出优化营商环境，加强关键领域改革

　　以盘活资源要素和完善机制为抓手，按照自治区优化营商环境大行动部署，打造良好营商环境，促进要素流动，持续激发市场活力和社会创造力。

　　依法盘活土地资源，改革国有资产和投融资管理体制。盘活西湾片区、常山片区、江山片区等十个片区的存量土地资源，促进土地资源优化配置。开展经开区、高新区建设用地统一征用统一安置。调查了解清楚国有资产存量，盘活存量国有资产，优化国有资产配置，推动国有资产集中统一监管。综合运用政府债券、企业债券、融资租赁、政府购买服务等融资方式，扩大金融有效供给，发挥财政资金放大作用和乘数效应。适应国家产业扶持方式的转变，用足用好各类产业投资基金，壮大发展产业基金规模。建立多元融资制度，推动PPP项目，增强县（市、区）政府投融资平台的融资能力。

　　抓好关键领域改革，完善工作体制机制。继续推动自治区党委、政府关于《东兴重点开发开放试验区管理体制改革总体方案》的贯彻落实，巩固深化构建开放型经济新体制综合试点试验和沿边金融综合改革试验工作。深化经开区、高新区机构设置、全员聘用、竞争上岗方面的改革。用足先行先试政策，加强人才小高地建设。完善国有平台公司的经营业绩评价考核机制。改革完善市本级与港口区、防城区的事权与支出责任划分，调动城区积极性。抓好企沙镇行政管理体制改革。启动公交经营体制改革，新增、调整公交线路，更加方便群众出行。依法规范市本级财政投资评审报告审批流程、建设用地用海审批管理、建设用地批后管理、财政性投资项目工程变更管理等工作制度和运行流程，提高效率，防止浪费，防范风险，杜绝腐败。

　　多渠道降低要素成本。抓好国家6项减税和自治区降成本“41条”“新28条”政策措施的落实。用好跨境劳务合作政策，帮助企业降低劳动力成本。加强对特许经营领域的监管，防止要素价格不合理上涨。推进电力直接交易，完善管道燃气定价机制，多渠道降低水电气等生产经营成本，今年在降低企业大宗用水价格方面要明显见效。

　　切实防范化解重大风险。完善金融机构监管制度，加强对小额贷款公司、融资性担保公司等七类地方金融机构的监管，提高群众金融风险意识，坚决打击非法集资、金融传销、洗钱等涉众型、风险型金融犯罪活动。健全金融监管体系，加强重点督查和审计，切实做好风险点管控，从小处着手，防微杜渐，促进经济安全稳健运行。

　　深化“放管服”改革。发挥市行政审批局职能，完善权力清单、责任清单，建立负面清单。实施“多证合一”“一照通”审批改革，强化事中事后监管。探索“证照分离”改革，推进企业登记全程电子化和电子营业执照应用，促进投资创业快捷化、企业服务便利化。进一步优化行政审批流程，推进网上审批，提升项目并联审批信息平台运行效率，实现政务服务便捷化。

　　三、加强政府自身建设

　　以创新政府工作机制为抓手，增强专业能力和专业精神，强化责任担当，狠抓工作落实，打造廉洁高效法治政府。

　　强化制度建设。开展“学文件懂业务用政策”活动，全面提高运用政策的能力水平。加强绩效管理，完善奖惩制度，对各县（市、区）和市直部门工作在全区进度排位实行预警预告制度，倒逼工作落实和终端见效。继续实行绩效管理1+4制度，推动各项工作提速提质提效。改进文风会风，推行精文简会。少开大会，多开小会，少开室内会，多开现场会、联席会、周例会，开会要议而决，决而行，行而果。倡导一线工作法，问题在一线发现，矛盾在一线解决，协调在一线进行，效果在一线体现，作风在一线锤炼，干部在一线成长。政府部门原则上不下发指导意见的文件，要多出实施方案，方案要明确完成时间、责任内容。改进征求意见的方式方法，再造办文流程，缩短办文时间。完善集体领导与个人分工负责制，突出权责对等，推动管理扁平化、极简化、专业化，确保政府机关高效运转。

　　提高行政执行力。今年要通过开展“产业提升年”“项目提速年”“城乡提质年”三个年活动，思想再解放，改革再深入，工作再抓实，不断提高政府工作执行力，促进我市产业、项目和城乡建设等重点关键工作取得新的突破。要培养专业能力和专业精神，全面增强学习能力、政治领导能力、改革创新能力、科学发展能力、依法执政能力、群众工作能力、狠抓落实能力、驾驭风险能力八种本领，建设高素质专业化干部队伍，为推动防城港加快发展做好充分的思想准备、知识准备和能力准备。

　　全面推进依法行政。加强对宪法的学习和宣传，围绕服务大局，推进政府立法工作创新发展。健全决策机制，提高行政决策科学化、民主化、法制化水平。加强重大决策第三方评估，严格行政决策责任追究。推动决策、执行、管理、服务和结果公开，提升政府透明度。加快城市综合执法体制改革，推行跨部门、跨领域综合执法，推动行政执法权向乡镇延伸。做好“七五”普法工作，落实“谁执法、谁普法”责任制，推进和规范乡村（社区）法律顾问制度。自觉接受人大法律监督、工作监督和政协民主监督，认真办理人大代表议案、建议和政协委员提案。

　　大兴调查研究之风。没有调查就没有发言权，要力戒形式主义、官僚主义，紧扣贯彻落实党的十九大精神，紧扣重大决策部署，紧扣人民群众生产生活，紧扣经济社会发展的热点难点问题，放下架子、扑下身子，深入田间地头和厂矿车间，全面深入听民声、察民情、解民困。

　　加强廉政建设。把全面从严治党落实到政府工作的各个方面，不断提高政府治理能力现代化水平。坚持把纪律和规矩挺在前面，持之以恒落实中央八项规定精神，坚决反对“四风”，强化监察、审计监督作用，对公共资金、公共资源、国有资产和领导干部履行经济责任情况实施监督全覆盖。深入推进党风廉政建设和反腐败工作，营造风清气正、干事创业的良好氛围。

　　各位代表！昂首挺进新时代，聚力谱写新篇章！我们要更加紧密团结在以习近平同志为核心的党中央周围，在市委的坚强领导下，紧紧依靠全市各族人民，不驰于空想，不骛于虚声，勇立潮头，砥砺前行，奋力谱写新时代防城港发展新篇章，以优异成绩迎接防城港建市25周年和建港50周年、迎接改革开放40周年和自治区成立60周年！
